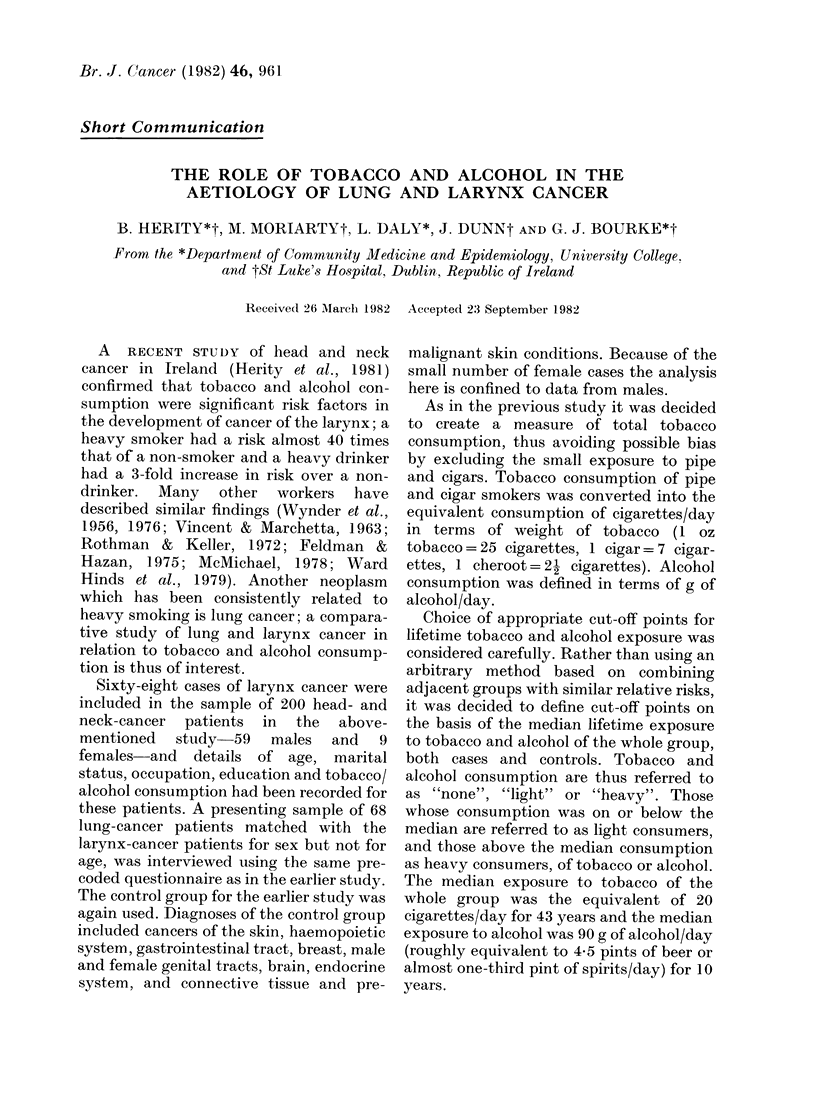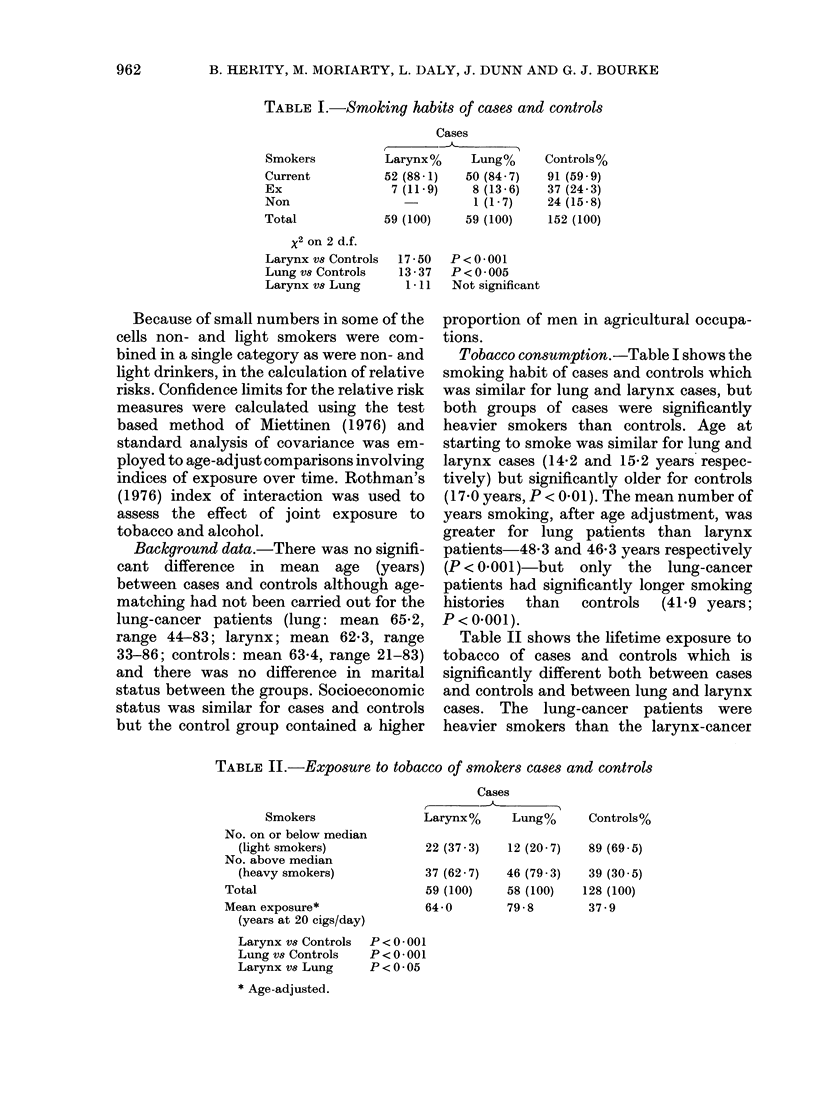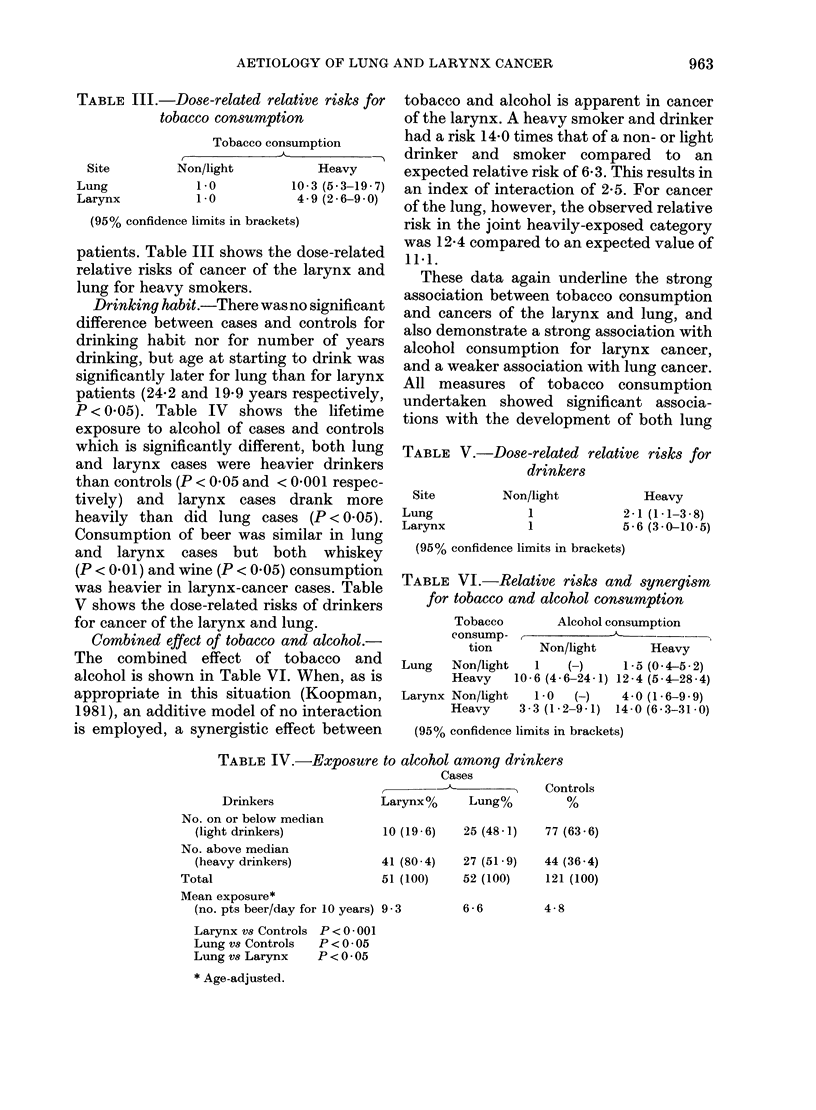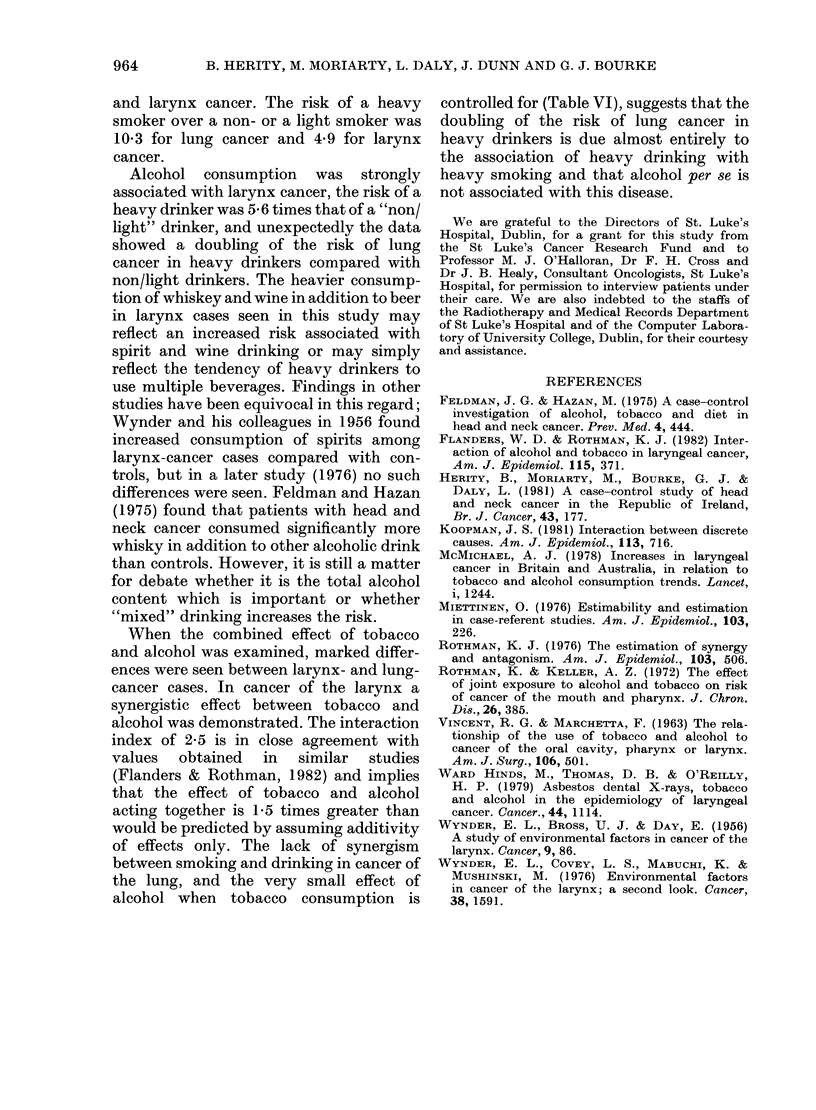# The role of tobacco and alcohol in the aetiology of lung and larynx cancer.

**DOI:** 10.1038/bjc.1982.308

**Published:** 1982-12

**Authors:** B. Herity, M. Moriarty, L. Daly, J. Dunn, G. J. Bourke


*Br. J. Cancer* (1982) **46**, 961

***Short Communication***

### THE ROLE OF TOBACCO AND ALCOHOL IN THE AETIOLOGY OF LUNG AND LARYNX CANCER

B. HERITY*†, M. MORIARTY†, L. DALY*, J. DUNN† AND G. J. BOURKE*†

*From the *Department of Community Medicine and Epidemiology, University College, and †St Luke's Hospital, Dublin, Republic of Ireland*

Received 26 March 1982 Accepted 23 September 1982

A RECENT STUDY of head and neck cancer in Ireland (Herity *et al.*, 1981) confirmed that tobacco and alcohol consumption were significant risk factors in the development of cancer of the larynx; a heavy smoker had a risk almost 40 times that of a non-smoker and a heavy drinker had a 3-fold increase in risk over a non-drinker. Many other workers have described similar findings (Wynder *et al.*, 1956, 1976; Vincent & Marchetta, 1963; Rothman & Keller, 1972; Feldman & Hazan, 1975; McMichael, 1978; Ward Hinds *et al.*, 1979). Another neoplasm which has been consistently related to heavy smoking is lung cancer; a comparative study of lung and larynx cancer in relation to tobacco and alcohol consumption is thus of interest.

Sixty-eight cases of larynx cancer were included in the sample of 200 head- and neck-cancer patients in the above-mentioned study—59 males and 9 females—and details of age, marital status, occupation, education and tobacco/alcohol consumption had been recorded for these patients. A presenting sample of 68 lung-cancer patients matched with the larynx-cancer patients for sex but not for age, was interviewed using the same pre-coded questionnaire as in the earlier study. The control group for the earlier study was again used. Diagnoses of the control group included cancers of the skin, haemopoietic system, gastrointestinal tract, breast, male and female genital tracts, brain, endocrine system, and connective tissue and pre-malignant skin conditions. Because of the small number of female cases the analysis here is confined to data from males.

As in the previous study it was decided to create a measure of total tobacco consumption, thus avoiding possible bias by excluding the small exposure to pipe and cigars. Tobacco consumption of pipe and cigar smokers was converted into the equivalent consumption of cigarettes/day in terms of weight of tobacco (1 oz tobacco = 25 cigarettes, 1 cigar = 7 cigarettes, 1 cheroot = $2\frac{1}{2}$ cigarettes). Alcohol consumption was defined in terms of g of alcohol/day.

Choice of appropriate cut-off points for lifetime tobacco and alcohol exposure was considered carefully. Rather than using an arbitrary method based on combining adjacent groups with similar relative risks, it was decided to define cut-off points on the basis of the median lifetime exposure to tobacco and alcohol of the whole group, both cases and controls. Tobacco and alcohol consumption are thus referred to as "none", "light" or "heavy". Those whose consumption was on or below the median are referred to as light consumers, and those above the median consumption as heavy consumers, of tobacco or alcohol. The median exposure to tobacco of the whole group was the equivalent of 20 cigarettes/day for 43 years and the median exposure to alcohol was 90 g of alcohol/day (roughly equivalent to 4·5 pints of beer or almost one-third pint of spirits/day) for 10 years.

TABLE I.—*Smoking habits of cases and controls*

| | Cases | | |
|---|---|---|---|
| Smokers | Larynx% | Lung% | Controls% |
| Current | 52 (88·1) | 50 (84·7) | 91 (59·9) |
| Ex | 7 (11·9) | 8 (13·6) | 37 (24·3) |
| Non | — | 1 (1·7) | 24 (15·8) |
| Total | 59 (100) | 59 (100) | 152 (100) |

| $\chi^2$ on 2 d.f. | | |
|---|---|---|
| Larynx *vs* Controls | 17·50 | $P<0.001$ |
| Lung *vs* Controls | 13·37 | $P<0.005$ |
| Larynx *vs* Lung | 1·11 | Not significant |

Because of small numbers in some of the cells non- and light smokers were combined in a single category as were non- and light drinkers, in the calculation of relative risks. Confidence limits for the relative risk measures were calculated using the test based method of Miettinen (1976) and standard analysis of covariance was employed to age-adjust comparisons involving indices of exposure over time. Rothman's (1976) index of interaction was used to assess the effect of joint exposure to tobacco and alcohol.

*Background data.*—There was no significant difference in mean age (years) between cases and controls although age-matching had not been carried out for the lung-cancer patients (lung: mean 65·2, range 44–83; larynx; mean 62·3, range 33–86; controls: mean 63·4, range 21–83) and there was no difference in marital status between the groups. Socioeconomic status was similar for cases and controls but the control group contained a higher proportion of men in agricultural occupations.

*Tobacco consumption.*—Table I shows the smoking habit of cases and controls which was similar for lung and larynx cases, but both groups of cases were significantly heavier smokers than controls. Age at starting to smoke was similar for lung and larynx cases (14·2 and 15·2 years respectively) but significantly older for controls (17·0 years, $P < 0.01$). The mean number of years smoking, after age adjustment, was greater for lung patients than larynx patients—48·3 and 46·3 years respectively ($P < 0.001$)—but only the lung-cancer patients had significantly longer smoking histories than controls (41·9 years; $P < 0.001$).

Table II shows the lifetime exposure to tobacco of cases and controls which is significantly different both between cases and controls and between lung and larynx cases. The lung-cancer patients were heavier smokers than the larynx-cancer

TABLE II.—*Exposure to tobacco of smokers cases and controls*

| | Cases | | |
|---|---|---|---|
| Smokers | Larynx% | Lung% | Controls% |
| No. on or below median (light smokers) | 22 (37·3) | 12 (20·7) | 89 (69·5) |
| No. above median (heavy smokers) | 37 (62·7) | 46 (79·3) | 39 (30·5) |
| Total | 59 (100) | 58 (100) | 128 (100) |
| Mean exposure* (years at 20 cigs/day) | 64·0 | 79·8 | 37·9 |

| | |
|---|---|
| Larynx *vs* Controls | $P<0.001$ |
| Lung *vs* Controls | $P<0.001$ |
| Larynx *vs* Lung | $P<0.05$ |

* Age-adjusted.

TABLE III.—*Dose-related relative risks for tobacco consumption*

| Site | Tobacco consumption: Non/light | Heavy |
|---|---|---|
| Lung | 1·0 | 10·3 (5·3–19·7) |
| Larynx | 1·0 | 4·9 (2·6–9·0) |

(95% confidence limits in brackets)

patients. Table III shows the dose-related relative risks of cancer of the larynx and lung for heavy smokers.

*Drinking habit.*—There was no significant difference between cases and controls for drinking habit nor for number of years drinking, but age at starting to drink was significantly later for lung than for larynx patients (24·2 and 19·9 years respectively, $P < 0·05$). Table IV shows the lifetime exposure to alcohol of cases and controls which is significantly different, both lung and larynx cases were heavier drinkers than controls ($P < 0·05$ and $< 0·001$ respectively) and larynx cases drank more heavily than did lung cases ($P < 0·05$). Consumption of beer was similar in lung and larynx cases but both whiskey ($P < 0·01$) and wine ($P < 0·05$) consumption was heavier in larynx-cancer cases. Table V shows the dose-related risks of drinkers for cancer of the larynx and lung.

*Combined effect of tobacco and alcohol.*—The combined effect of tobacco and alcohol is shown in Table VI. When, as is appropriate in this situation (Koopman, 1981), an additive model of no interaction is employed, a synergistic effect between tobacco and alcohol is apparent in cancer of the larynx. A heavy smoker and drinker had a risk 14·0 times that of a non- or light drinker and smoker compared to an expected relative risk of 6·3. This results in an index of interaction of 2·5. For cancer of the lung, however, the observed relative risk in the joint heavily-exposed category was 12·4 compared to an expected value of 11·1.

These data again underline the strong association between tobacco consumption and cancers of the larynx and lung, and also demonstrate a strong association with alcohol consumption for larynx cancer, and a weaker association with lung cancer. All measures of tobacco consumption undertaken showed significant associations with the development of both lung

TABLE V.—*Dose-related relative risks for drinkers*

| Site | Non/light | Heavy |
|---|---|---|
| Lung | 1 | 2·1 (1·1–3·8) |
| Larynx | 1 | 5·6 (3·0–10·5) |

(95% confidence limits in brackets)

TABLE VI.—*Relative risks and synergism for tobacco and alcohol consumption*

| | Tobacco consumption | Alcohol consumption: Non/light | Heavy |
|---|---|---|---|
| Lung | Non/light | 1 (–) | 1·5 (0·4–5·2) |
| | Heavy | 10·6 (4·6–24·1) | 12·4 (5·4–28·4) |
| Larynx | Non/light | 1·0 (–) | 4·0 (1·6–9·9) |
| | Heavy | 3·3 (1·2–9·1) | 14·0 (6·3–31·0) |

(95% confidence limits in brackets)

TABLE IV.—*Exposure to alcohol among drinkers*

| Drinkers | Cases: Larynx% | Lung% | Controls % |
|---|---|---|---|
| No. on or below median (light drinkers) | 10 (19·6) | 25 (48·1) | 77 (63·6) |
| No. above median (heavy drinkers) | 41 (80·4) | 27 (51·9) | 44 (36·4) |
| Total | 51 (100) | 52 (100) | 121 (100) |
| Mean exposure* (no. pts beer/day for 10 years) | 9·3 | 6·6 | 4·8 |

Larynx *vs* Controls $P < 0·001$
Lung *vs* Controls $P < 0·05$
Lung *vs* Larynx $P < 0·05$

* Age-adjusted.

and larynx cancer. The risk of a heavy smoker over a non- or a light smoker was 10·3 for lung cancer and 4·9 for larynx cancer.

Alcohol consumption was strongly associated with larynx cancer, the risk of a heavy drinker was 5·6 times that of a "non/light" drinker, and unexpectedly the data showed a doubling of the risk of lung cancer in heavy drinkers compared with non/light drinkers. The heavier consumption of whiskey and wine in addition to beer in larynx cases seen in this study may reflect an increased risk associated with spirit and wine drinking or may simply reflect the tendency of heavy drinkers to use multiple beverages. Findings in other studies have been equivocal in this regard; Wynder and his colleagues in 1956 found increased consumption of spirits among larynx-cancer cases compared with controls, but in a later study (1976) no such differences were seen. Feldman and Hazan (1975) found that patients with head and neck cancer consumed significantly more whisky in addition to other alcoholic drink than controls. However, it is still a matter for debate whether it is the total alcohol content which is important or whether "mixed" drinking increases the risk.

When the combined effect of tobacco and alcohol was examined, marked differences were seen between larynx- and lung-cancer cases. In cancer of the larynx a synergistic effect between tobacco and alcohol was demonstrated. The interaction index of 2·5 is in close agreement with values obtained in similar studies (Flanders & Rothman, 1982) and implies that the effect of tobacco and alcohol acting together is 1·5 times greater than would be predicted by assuming additivity of effects only. The lack of synergism between smoking and drinking in cancer of the lung, and the very small effect of alcohol when tobacco consumption is controlled for (Table VI), suggests that the doubling of the risk of lung cancer in heavy drinkers is due almost entirely to the association of heavy drinking with heavy smoking and that alcohol *per se* is not associated with this disease.

We are grateful to the Directors of St. Luke's Hospital, Dublin, for a grant for this study from the St Luke's Cancer Research Fund and to Professor M. J. O'Halloran, Dr F. H. Cross and Dr J. B. Healy, Consultant Oncologists, St Luke's Hospital, for permission to interview patients under their care. We are also indebted to the staffs of the Radiotherapy and Medical Records Department of St Luke's Hospital and of the Computer Laboratory of University College, Dublin, for their courtesy and assistance.

### REFERENCES

FELDMAN, J. G. & HAZAN, M. (1975) A case–control investigation of alcohol, tobacco and diet in head and neck cancer. *Prev. Med.* **4**, 444.

FLANDERS, W. D. & ROTHMAN, K. J. (1982) Interaction of alcohol and tobacco in laryngeal cancer, *Am. J. Epidemiol.* **115**, 371.

HERITY, B., MORIARTY, M., BOURKE, G. J. & DALY, L. (1981) A case–control study of head and neck cancer in the Republic of Ireland, *Br. J. Cancer*, **43**, 177.

KOOPMAN, J. S. (1981) Interaction between discrete causes. *Am. J. Epidemiol.*, **113**, 716.

MCMICHAEL, A. J. (1978) Increases in laryngeal cancer in Britain and Australia, in relation to tobacco and alcohol consumption trends. *Lancet*, i, 1244.

MIETTINEN, O. (1976) Estimability and estimation in case-referent studies. *Am. J. Epidemiol.*, **103**, 226.

ROTHMAN, K. J. (1976) The estimation of synergy and antagonism. *Am. J. Epidemiol.*, **103**, 506.

ROTHMAN, K. & KELLER, A. Z. (1972) The effect of joint exposure to alcohol and tobacco on risk of cancer of the mouth and pharynx. *J. Chron. Dis.*, **26**, 385.

VINCENT, R. G. & MARCHETTA, F. (1963) The relationship of the use of tobacco and alcohol to cancer of the oral cavity, pharynx or larynx. *Am. J. Surg.*, **106**, 501.

WARD HINDS, M., THOMAS, D. B. & O'REILLY, H. P. (1979) Asbestos dental X-rays, tobacco and alcohol in the epidemiology of laryngeal cancer. *Cancer.*, **44**, 1114.

WYNDER, E. L., BROSS, U. J. & DAY, E. (1956) A study of environmental factors in cancer of the larynx. *Cancer*, **9**, 86.

WYNDER, E. L., COVEY, L. S., MABUCHI, K. & MUSHINSKI, M. (1976) Environmental factors in cancer of the larynx; a second look. *Cancer*, **38**, 1591.